# Regularization with Metric Double Integrals of Functions with Values in a Set of Vectors

**DOI:** 10.1007/s10851-018-00869-6

**Published:** 2019-02-07

**Authors:** René Ciak, Melanie Melching, Otmar Scherzer

**Affiliations:** 1 Computational Science Center, University of Vienna, Oskar-Morgenstern-Platz 1, 1090 Vienna, Austria; 2 Johann Radon Institute for Computational and Applied Mathematics (RICAM), Altenbergstraße 69, 4040 Linz, Austria

**Keywords:** Regularization, Manifold-valued data, Non-convex, Metric, Double integral, Fractional Sobolev space, Bounded variation

## Abstract

We present an approach for variational regularization of inverse and imaging problems for recovering functions with values in a set of vectors. We introduce regularization functionals, which are derivative-free double integrals of such functions. These regularization functionals are motivated from double integrals, which approximate Sobolev semi-norms of intensity functions. These were introduced in Bourgain et al. (Another look at Sobolev spaces. In: Menaldi, Rofman, Sulem (eds) Optimal control and partial differential equations-innovations and applications: in honor of professor Alain Bensoussan’s 60th anniversary, IOS Press, Amsterdam, pp 439–455, 2001). For the proposed regularization functionals, we prove existence of minimizers as well as a stability and convergence result for functions with values in a set of vectors.

## Introduction

Functions with values in a (nonlinear) subset of a vector space appear in several applications of imaging and in inverse problems, e.g.,

- *Interferometric Synthetic Aperture Radar (InSAR)* is a technique used in remote sensing and geodesy to generate, for example, digital elevation maps of the earth’s surface. InSAR images represent phase differences of waves between two or more SAR images, cf. [44, 53]. Therefore, InSAR data are functions $f:\Omega \rightarrow {\mathbb {S}}^1\subseteq {\mathbb {R}}^2$. The pointwise function values are on the ${\mathbb {S}}^1$, which is considered embedded into ${\mathbb {R}}^2$.
- A *color image* can be represented as a function in *HSV* space (hue, saturation, value) (see, e.g., [48]). Color images are then described as functions $f:\Omega \rightarrow K \subseteq {\mathbb {R}}^3$. Here $\Omega$ is a plane in ${\mathbb {R}}^2$, the image domain, and *K* (representing the HSV space) is a cone in three-dimensional space ${\mathbb {R}}^3$.
- Estimation of the *foliage angle distribution* has been considered, for instance, in [39, 51]. Therefore, the imaging function is from $\Omega \subset {\mathbb {R}}^2$, a part of the Earth’s surface, into $\mathbb {S}^2 \subseteq {\mathbb {R}}^3$, representing foliage angle orientation.
- Estimation of functions with values in $SO(3) \subseteq {\mathbb {R}}^{3 \times 3}$. Such problems appear in *Cryo-Electron Microscopy* (see, for instance, [38, 58, 61]).

We emphasize that we are analyzing *vector*-, *matrix*-, *tensor*- *valued* functions, where pointwise function evaluations belong to some given (sub)set, but are always *elements* of the underlying vector space. This should not be confused with set-valued functions, where every function evaluation can be a set.

Inverse problems and imaging tasks, such as the ones mentioned above, might be unstable, or even worse, the solution could be ambiguous. Therefore, numerical algorithms for imaging need to be *regularizing* to obtain approximations of the desired solution in a stable manner. Consider the operator equation1.1where we assume that only (noisy) measurement data $v^\delta$ of $v^0$ become available. In this paper the method of choice is *variational regularization* which consists in calculating a minimizer of the variational regularization functional1.2Here

- *w* is an element of the *set* of admissible functions.
is an operator modeling the image formation process (except the noise).
- $\mathcal {D}$ is called the *data* or *fidelity term*, which is used to compare a pair of data in the image domain, that is to quantify the difference of the two data sets.
- $\mathcal {R}$ is called *regularization functional*, which is used to impose certain properties onto a minimizer of the regularization functional $\mathcal {F}$.
- $\alpha > 0$ is called *regularization parameter* and provides a trade off between stability and approximation properties of the minimizer of the regularization functional $\mathcal {F}$.
- $v^\delta$ denotes measurement data, which we consider noisy.
- $v^0$ denotes the exact data, which we assume to be not necessarily available.

The main objective of this paper is to introduce a general class of regularization functionals for functions with values in a set of vectors. In order to motivate our proposed class of regularization functionals, we review a class of regularization functionals appropriate for analyzing *intensity data*.

### Variational Regularization for Reconstruction of Intensity Data

Opposite to what we consider in the present paper, most commonly, imaging data *v* and admissible functions *w*, respectively, are considered to be representable as *intensity functions*. That is, they are functions from some subset $\Omega$ of an Euclidean space with *real values*.

In such a situation, the most widely used regularization functionals use regularization terms consisting of powers of Sobolev (see [12, 15, 16]) or total variation semi-norms [54]. It is common to speak about *Tikhonov regularization* (see, for instance, [59]) when the data term and the regularization functional are squared Hilbert space norms, respectively. For the *Rudin, Osher, Fatemi (ROF)* regularization [54], also known as total variation regularization, the data term is the squared $L^2$-norm and $\mathcal {R}(w) = |w|_{TV}$ is the total variation semi-norm. Nonlocal regularization operators based on the generalized nonlocal gradient are used in [35].

Other widely used regularization functionals are *sparsity promoting* [22, 41], *Besov space norms* [42, 46] and anisotropic regularization norms [47, 56]. Aside from various regularization terms, there also have been proposed different fidelity terms other than quadratic norm fidelities, like the *p*-th powers of $\ell ^p$ and $L^p$-norms of the differences of *F*(*w*) and *v* , [55, 57], maximum entropy [26, 28] and Kullback–Leibler divergence [52] (see [50] for some reference work).

Our work utilizes results from the seminal paper of Bourgain, Brézis and Mironescu [14], which provides an equivalent *derivative-free* characterization of Sobolev spaces and the space , the space of functions of bounded total variation, which consequently, in this context, was analyzed in Dávila and Ponce [23, 49], respectively. It is shown in [14, Theorems 2 and 3’] and [23, Theorem 1] that when $(\rho _\varepsilon )_{\varepsilon > 0}$ is a suitable sequence of nonnegative, radially symmetric, radially decreasing mollifiers, then1.3Hence, $\tilde{{\mathcal {R}}}_\varepsilon$ approximates powers of Sobolev semi-norms and the total variation semi-norm, respectively. Variational imaging, consisting in minimization of $\mathcal {F}$ from Eq. 1.2 with ${\mathcal {R}}$ replaced by $\tilde{{\mathcal {R}}}_\varepsilon$, has been considered in [3, 11].

### Regularization of Functions with Values in a Set of Vectors

In this paper we generalize the derivative-free characterization of Sobolev spaces and functions of bounded variation to functions $u:\Omega \rightarrow K$, where *K* is some set of vectors, and use these functionals for variational regularization. The applications we have in mind contain that *K* is a closed subset of ${\mathbb {R}}^M$ (for instance, HSV data) with nonzero measure, or that *K* is a submanifold (for instance, InSAR data).

The reconstruction of manifold-valued data with variational regularization methods has already been subject to intensive research (see, for instance, [4, 17–19, 40, 62]). The variational approaches mentioned above use regularization and fidelity functionals based on Sobolev and TV semi-norms: a total variation regularizer for cyclic data on ${\mathbb {S}}^1$ was introduced in [18, 19], see also [7, 9, 10]. In [4, 6] combined first- and second-order differences and derivatives were used for regularization to restore manifold-valued data. The later mentioned papers, however, are formulated in a finite-dimensional setting, opposed to ours, which is considered in an infinite-dimensional setting. Algorithms for total variation minimization problems, including half-quadratic minimization and nonlocal patch-based methods, are given, for example, in [4, 5, 8] as well as in [37, 43]. On the theoretical side the total variation of functions with values in a manifold was investigated by Giaquinta and Mucci using the theory of Cartesian currents in [33, 34], and earlier [32] if the manifold is ${\mathbb {S}}^1$.

### Content and Particular Achievements of the Paper

The contribution of this paper is to introduce and analytically analyze double integral regularization functionals for reconstructing functions with values in a set of vectors, generalizing functionals of the form Eq. 1.3. Moreover, we develop and analyze fidelity terms for comparing manifold-valued data. Summing these two terms provides a new class of regularization functionals of the form Eq. 1.2 for reconstructing manifold-valued data.

When analyzing our functionals, we encounter several differences to existing regularization theory (compare Sect. 2):

(i) The *admissible functions*, where we minimize the regularization functional on, do form only a *set* but *not* a *linear* space. As a consequence, well-posedness of the variational method (that is, existence of a minimizer of the energy functional) cannot directly be proven by applying standard direct methods in the Calculus of Variations [20, 21].
(ii) The regularization functionals are defined via metrics and not norms, see Sect. 3.
(iii) In general, the fidelity terms are *non-convex*. Stability and convergence results are proven in Sect. 4.

The model is validated in Sect. 6 where we present numerical results for denoising and inpainting of data of InSAR type.

## Setting

In the following we introduce the basic notation and the set of admissible functions which we are regularizing on.

### Assumption 2.1

All along this paper, we assume that

- $$p_1, p_2 \in [1, +\infty )$$, $$s \in (0,1]$$,
- $\Omega _1, \Omega _2 \subseteq {\mathbb {R}}^N$ are nonempty, bounded and connected open sets with Lipschitz boundary, respectively,
- $$k \in [0,N]$$,
- $K_1 \subseteq {\mathbb {R}}^{M_1}, K_2 \subseteq {\mathbb {R}}^{M_2}$ are nonempty and closed subsets of ${\mathbb {R}}^{M_1}$ and ${\mathbb {R}}^{M_2}$, respectively.

Moreover,

and $\Vert \cdot \Vert _{{\mathbb {R}}^{M_i}}, \ i=1,2,$ are the Euclidean norms on ${\mathbb {R}}^N$ and ${\mathbb {R}}^{M_i}$, respectively.
denotes the Euclidean distance on ${\mathbb {R}}^{M_i}$ for $i=1,2$ and
denote arbitrary metrics on $K_i$, which fulfill for $i=1$ and $i=2$
  ,
  - $\,{\mathrm {d}}_i$ is continuous with respect to , meaning that for a sequence  in $K_i \subseteq {\mathbb {R}}^{M_i}$ converging to some $a \in K_i$ we also have .
  In particular, this assumption is valid if the metric $d_i$ is equivalent to . When the set $K_i, \ i=1,2$, is a suitable complete submanifold of ${\mathbb {R}}^{M_i}$, it seems natural to choose $d_i$ as the geodesic distance on the respective submanifolds.
- $(\rho _{\varepsilon })_{\varepsilon > 0}$ is a Dirac family of nonnegative, radially symmetric mollifiers, i.e., for every $\varepsilon > 0$ we have
  (i) $\rho _\varepsilon \in \mathcal {C}^{\infty }_{c}({\mathbb {R}}^N, {\mathbb {R}})$ is radially symmetric,
  (ii) $$\rho _\varepsilon \ge 0$$,
  (iii) $$\int \limits _{{\mathbb {R}}^N} \rho _\varepsilon (x) \,{\mathrm {d}}x= 1$$, and
  (iv) for all $\delta > 0$, .
  We demand further that, for every $\varepsilon > 0$,
  (v) there exists a $\tau > 0$ and $\eta _{\tau }> 0$ such that .
  This condition holds, e.g., if $\rho _{\varepsilon }$ is a radially decreasing continuous function with $\rho _{\varepsilon }(0) > 0$.
- When we write *p*, $\Omega$, *K*, *M*, then we mean $p_i$, $\Omega _i$, $K_i$, $M_i$, for either $i=1,2$. In the following we will often omit the subscript indices whenever possible.

### Example 2.2

Let $\hat{\rho } \in C_c^\infty ({\mathbb {R}},{\mathbb {R}}_+)$ be symmetric at 0, monotonically decreasing on $[0, \infty )$ and satisfy

$$\begin{aligned} \left| \mathbb {S}^{N-1}\right| \int _0^\infty \hat{t}^{N-1} \hat{\rho }\left( \hat{t}\right) \mathrm{d} \hat{t} = 1. \end{aligned}$$

Defining mappings $\rho _\varepsilon : {\mathbb {R}}^N \rightarrow {\mathbb {R}}$ byconstitutes then a family $(\rho _\varepsilon )_{\varepsilon > 0}$ which fulfills the above properties (i)–(v). Note here that

- by substitution $x = t \theta$ with $t > 0, \theta \in \mathbb {S}^{N-1}$ and $\hat{t}=\frac{t}{\varepsilon }$, 2.1 Here, $d\theta$ refers to the canonical spherical measure.
- Again by the same substitutions, taking into account that $\hat{\rho }$ has compact support, it follows for $\varepsilon > 0$ sufficiently small that 2.2

In the following we write down the basic spaces and sets, which will be used in the course of the paper.

### Definition 2.3

- The *Lebesgue–Bochner space* of ${\mathbb {R}}^M$-valued functions on $\Omega$ consists of the set  which is associated with the norm , given by
- Let $0< s < 1$. Then the *fractional Sobolev space* of order *s* can be defined (cf. [1]) as the set  equipped with the norm 2.3 where  is the semi-norm for , given by 2.4
- For $s = 1$ the Sobolev space $W^{1,p}(\Omega , {\mathbb {R}}^M)$ consists of all weakly differentiable functions in $L^1(\Omega ,{\mathbb {R}}^M)$ for which  where $\nabla w$ is the weak Jacobian of *w*.
- Moreover, we recall one possible definition of the space  from [2], which consists of all Lebesgue–Borel measurable functions $w:\Omega \rightarrow {\mathbb {R}}^M$ for which  where  where $\left\| \varphi (x)\right\| _F$ is the Frobenius-norm of the matrix $\varphi (x)$ and $\text {Div}\varphi = (\text {div} \varphi _1, \dots , \text {div} \varphi _M)^\text {T}$ denotes the row–wise formed divergence of $\varphi$.

### Lemma 2.4

Let $0 < s \le 1$ and $p \in [1,\infty )$, then  and the embedding is compact. Moreover, the embedding  is compact for all

$$\begin{aligned} 1 \le p < 1^* :={\left\{ \begin{array}{ll} +\infty &{}\text{ if } N = 1 \\ \frac{N}{N-1} &{}\text{ otherwise } \end{array}\right. }. \end{aligned}$$

### Proof

The first result can be found in [24] for $0< s < 1$ and in [29] for $s = 1$. The second assertion is stated in [2]. $\square$

### Remark 2.5

Let Assumption 2.1 hold. We recall some basic properties of weak convergence in , $W^{1,p}(\Omega , {\mathbb {R}}^M)$ and weak* convergence in  (see, for instance, [1, 2]):

- Let $p > 1$, $s\in (0,1]$ and assume that $(w_n)_{n \in {\mathbb {N}}}$ is bounded in . Then there exists a subsequence $(w_{n_k})_{k \in {\mathbb {N}}}$ which converges weakly in .
- Assume that $(w_n)_{n \in {\mathbb {N}}}$ is bounded in . Then there exists a subsequence $(w_{n_k})_{k \in {\mathbb {N}}}$ which converges weakly* in .

Before introducing the regularization functional, which we investigate theoretically and numerically, we give the definition of some sets of (equivalence classes of) admissible functions.

### Definition 2.6

For $0 < s \le 1$, $p \ge 1$ and a nonempty closed subset $K \subseteq {\mathbb {R}}^M$, we defineand equip each of these (in general nonlinear) sets with some subspace topology:

is associated with the strong -topology,
is associated with the weak -topology, and
is associated with the weak* -topology.

Moreover, we define2.5Consistently, $W(\Omega ,K)$

- is associated with the weak -topology in the case $p \in (1, \infty )$ and $s \in (0,1]$ and
- with the weak* -topology when $p=1$ and $s=1$.

When we speak aboutand mean weak convergence on $W^{s,p}(\Omega ,K)$ and weak* convergence on , respectively.

### Remark 2.7

- In general  and  are sets which do not form a linear space.
- If $K = {\mathbb {S}}^1$, then  as occurred in [13].
- For an embedded manifold *K*, the dimension of the manifold is not necessarily identical with the space dimension of ${\mathbb {R}}^M$. For instance, if $K = {\mathbb {S}}^1\subseteq {\mathbb {R}}^2$, then the dimension of ${\mathbb {S}}^1$ is 1 and $M=2$.

The following lemma shows that $W(\Omega ,K)$ is a sequentially closed subset of .

### Lemma 2.8

(Sequential closedness of ${{W}}({{\Omega }},{{K}})$ and ${{L}}^{{{p}}}({{\Omega }}, {{K}})$)

(i) Let  and $(w_n)_{n\in {\mathbb {N}}}$ be a sequence in  with $w_n \overset{W(\Omega , {\mathbb {R}}^M)}{\longrightarrow } w_*$ as $n \rightarrow \infty$. Then  and  in .
(ii) Let  and $(v_n)_{n \in {\mathbb {N}}}$ be a sequence in  with $v_n \rightarrow v_*$ in  as $n \rightarrow \infty$. Then  and there is some subsequence $(v_{n_k})_{k \in {\mathbb {N}}}$ which converges to $v_*$ pointwise almost everywhere, i.e., $v_{n_k}(x) \rightarrow v_*(x)$ as $k \rightarrow \infty$ for almost every $x \in \Omega$.

### Proof

For the proof of the second part, cf. [27], Chapter VI, Corollary 2.7, take into account the closedness of $K \subseteq {\mathbb {R}}^M$. The proof of the first part follows from standard convergence arguments in ,  and , respectively, using the embeddings from Lemma 2.4, an argument on subsequences and part two. $\square$

### Remark 2.9

Lemma 2.4 along with Lemma 2.8 imply that  is compactly embedded in , where these sets are equipped with the bornology inherited from  and the topology inherited from , respectively.

In the following we postulate the assumptions on the operator  which will be used throughout the paper:

### Assumption 2.10

Let  be as in Eq. 2.5 and assume that  is an operator from  to .

We continue with the definition of our regularization functionals:

### Definition 2.11

Let Assumptions 2.1 and 2.10 hold. Moreover, let $\varepsilon > 0$ be fixed and let $\rho :=\rho _\varepsilon$ be a mollifier.

The regularization functional  is defined as follows2.6where

(i),
(ii) $$s \in (0,1]$$,
(iii) $\alpha \in (0, +\infty )$ is the regularization parameter,
(iv) $l \in \left\{ 0, 1\right\}$ is an indicator and
(v) $${\left\{ \begin{array}{ll} k \le N &{}\text{ if } W (\Omega _1, K_1) = W^{s,p_1}(\Omega _1, K_1), \ 0{<}s{<}1, \\ k=0 &{} \text{ if } W (\Omega _1, K_1) = W^{1,p_1}(\Omega _1, K_1)\text { or if }\\ &{}\quad W (\Omega _1, K_1) = BV(\Omega _1, K_1), \text { respectively.} \end{array}\right. }$$

Setting2.7and2.8Equation 2.6 can be expressed in compact form2.9For convenience we will often skip some of the super- or subscript and use compact notations like, e.g.,

### Remark 2.12

(i) $l = \left\{ 0,1\right\}$ is an indicator which allows to consider approximations of Sobolev semi-norms and double integral representations of the type of Bourgain et al. [14] in a uniform manner.
  when $k=0$, $s=1$, $l=1$ and when $d_1$ is the Euclidean distance, we get the double integrals of the Bourgain et al.-form [14]. Compare with Eq. 1.3.
  When $d_1$ is the Euclidean distance, $k=N$ and $l=0$, we get Sobolev semi-norms.
  We expect a relation between the two classes of functionals for $l=0$ and $l=1$ as stated in Sect. 5.2.
(ii) When $d_1$ is the Euclidean distance then the second term in Eq. 2.6 is similar to the ones used in [3, 11, 14, 23, 49].

In the following we state basic properties of  and the functional .

### Proposition 2.13

Let Assumption 2.1 hold.

(i) Then the mapping    satisfies the metric axioms.
(ii) Let, in addition, Assumption 2.10 hold, assume that  and that both metrics $d_i$, $i=1,2$, are equivalent to , respectively. Then the functional  does not attain the value $+\infty$ on its domain .

### Proof

(i) The axioms of non-negativity, identity of indiscernibles and symmetry are fulfilled by  since  is a metric. To prove the triangle inequality, let $\phi ,\xi ,\nu \in L^{p_2}(\Omega _2, K_2)$. In the main case  Hölder’s inequality yields  meaning  If , the triangle inequality is trivially fulfilled.
  In the remaining case  applying the estimate $(a+b)^p \le 2^{p-1} (a^p + b^p)$, see, e.g., [55, Lemma 3.20], to  and  yields  implying the desired result.
(ii) We emphasize that  because every constant function $w(\cdot ) = a \in K_1$ belongs to  for $p_1 \in (1, \infty )$ and $s \in (0,1]$ as well as to  for $p_1 = 1$ and $s = 1$. Assume now that the metrics $d_i$ are equivalent to  for $i=1$ and $i=2$, respectively, so that we have an upper bound . We need to prove that  for every . Due to  for all  it is sufficient to show  for all .
  For  this is guaranteed by [49, Theorem 1.2].
  For  by [14, Theorem 1].
  For , $s \in (0,1)$, we distinguish between two cases.
    If , we have that  for $k \le N$ and hence  If , we can estimate  In summary adding yields . $\square$

## Existence

In order to prove existence of a minimizer of the functional , we apply the direct method in the Calculus of Variations (see, e.g., [20, 21]). To this end we verify continuity properties of  and , resp.  and apply them along with the sequential closedness of , already proven in Lemma 2.8.

In this context we point out some setting assumptions and their consequences on , resp.  and ${\mathcal {R}}$ in the following remark. For simplicity we assume $p :=p_1 = p_2 \in (1, \infty )$, $\Omega :=\Omega _1 = \Omega _2$ and .

### Remark 3.1

- The continuity of  with respect to  guarantees lower semicontinuity of  and .
- The inequality  carries over to the inequalities  for all , and  for all , allowing to transfer properties like coercivity from  to . Moreover, the extended real-valued metric space  stays related to the linear space  in terms of the topology and bornology induced by , resp. those inherited by .
- The closedness of $K \subseteq {\mathbb {R}}^M$ is crucial in showing that  is a sequentially closed subset of the linear space . This closedness property acts as a kind of replacement for the, a priori not available, notion of completeness with respect to the “space” .

For $l=0$, $k=N$ note in the latter item that equipping  with  and  does not even lead to an (extended real-valued) metric space, in contrast to the classical case .

We will use the following assumption:

### Assumption 3.2

Let Assumption 2.1 hold,  and let  and the associated topology be as defined in Eq. 2.5.

In addition we assume:

is well defined and sequentially continuous with respect to the specified topology on  and
- For every $t > 0$ and $\alpha > 0$, the level sets 3.1 are sequentially pre-compact subsets of $W(\Omega _1, {\mathbb {R}}^{M_1})$.
- There exists a $\bar{t} > 0$ such that  is nonempty.
- Only those  are considered which additionally fulfill .

### Remark 3.3

The third condition is sufficient to guarantee . In contrast, the condition , cf. Definition 2.11, might not be sufficient if $d_2$ is not equivalent to .

### Lemma 3.4

Let Assumption 3.2 hold. Then the mappings ,  and  have the following continuity properties:

(i) The mapping  is sequentially lower semi-continuous, i.e., whenever sequences ,  in  converge to  and , respectively, we have .
(ii) The functional  is sequentially lower semi-continuous, i.e., whenever a sequence $(w_n)_{n \in {\mathbb {N}}}$ in  converges to some  we have
(iii) The functional  is sequentially lower semi-continuous.

### Proof

(i) It is sufficient to show that for *every* pair of sequences ,  in  which converge to previously *fixed* elements  and , respectively, we can extract subsequences $(\phi _{n_j})_{j \in {\mathbb {N}}}$ and $(\nu _{n_j})_{j \in {\mathbb {N}}}$, respectively, with  To this end let $(\phi _n)_{n \in {\mathbb {N}}},(\nu _n)_{n \in {\mathbb {N}}}$ be some sequences in  with  and  in . Lemma 2.8 ensures that there exist subsequences $(\phi _{n_j})_{j \in {\mathbb {N}}}, (\nu _{n_j})_{j \in {\mathbb {N}}}$ converging to $\phi _*$ and $\nu _*$ pointwise almost everywhere, which in turn implies $\big (\phi _{n_j}(\cdot ), \nu _{n_j}(\cdot ) \big ) \rightarrow \big ( \phi _*(\cdot ), \nu _*(\cdot ) \big )$ pointwise almost everywhere. Therefrom, together with the continuity of  with respect to , cf. Sect. 2, we obtain by using the quadrangle inequality that  and hence  for almost every $x \in \Omega _2$. Applying Fatou’s lemma, we obtain
(ii) Let $(w_n)_{n \in {\mathbb {N}}}$ be a sequence in  with  as . By Lemma 2.8 there is a subsequence $(w_{n_j})_{j \in {\mathbb {N}}}$ which converges to $w_*$ both in  and pointwise almost everywhere. This further implies that  for almost every
  3.2 $$\begin{aligned} (x,y) \in \Omega _1 \times \Omega _1 \supseteq \{(x,y) \in \Omega _1 \times \Omega _1 : x \ne y \} =:A.\nonumber \\ \end{aligned}$$
  Defining  for all $j \in {\mathbb {N}}$ and  we thus have  for almost every $(x,y) \in \Omega _1 \times \Omega _1$. Applying Fatou’s lemma to the functions $f_j$ yields the assertion, due to the same reduction as in the proof of the first part.
(iii) It is sufficient to prove that the components  and  of  are sequentially lower semi-continuous. To prove that $\mathcal {G}$ is sequentially lower semi-continuous in every $w_* \in W(\Omega _1, K_1)$, let $(w_n)_{n \in {\mathbb {N}}}$ be a sequence in $W(\Omega _1, K_1)$ with  as . Assumption 3.2, ensuring the sequential continuity of , implies hence  in  as . By item *(i)* we thus obtain .
  ${\mathcal {R}}$ is sequentially lower semi-continuous by item *(ii)*.

$$\square $$

### Existence of Minimizers

The proof of the existence of a minimizer of  is along the lines of the proof in [55], taking into account Remark 3.1. We will need the following useful lemma, cf. [55], which links  and  for .

#### Lemma 3.5

It holdsfor every $w \in W(\Omega _1, K_1)$ and $v_\star , v_\diamond \in L^{p_2}(\Omega _2, K_2)$.

#### Proof

Using the fact that for $p \ge 1$ we have that $|a+b|^p \le 2^{p-1}(|a|^p + |b|^p), \ a,b \in {\mathbb {R}}\cup \{\infty \}$ and that  fulfills the triangle inequality, we obtain$\square$

#### Theorem 3.6

Let Assumption 3.2 hold. Then the functional  attains a minimizer.

#### Proof

We prove the existence of a minimizer via the direct method. We shortly write  for . Let $(w_n)_{n \in {\mathbb {N}}}$ be a sequence in $W(\Omega _1, K_1)$ with3.3The latter infimum is not $+\infty$, because  would imply also  due to Lemma 3.5, violating Assumption 3.2. In particular, there is some $c \in {\mathbb {R}}$ such that  for every $n \in {\mathbb {N}}$. Applying Lemma 3.5 yields  due to Assumption 3.2. Since the level set  is sequentially pre-compact with respect to the topology given to $W(\Omega _1, {\mathbb {R}}^{M_1})$ we get the existence of a subsequence $(w_{n_k})_{k \in {\mathbb {N}}}$ which converges to some $w_* \in W(\Omega _1, {\mathbb {R}}^{M_1})$, where actually $w_* \in W(\Omega _1, K_1)$ due to Lemma 2.8. Because  is sequentially lower semi-continuous, see Lemma 3.4, we have . Combining this with Eq. 3.3 we obtainIn particular, , meaning that $w_*$ is a minimizer of . $\square$

In the following we investigate two examples, which are relevant for the numerical examples in Sect. 6.

#### Example 3.7

We consider that $W(\Omega _1,K_1) = W^{s, p_1}(\Omega _1, K_1)$ with $p_1>1, \ 0< s < 1$ and fix $k = N$.

If the operator  is norm coercive in the sense that the implication3.4holds true for every sequence $(w_n)_{n \in {\mathbb {N}}}$ in $W^{s,p_1}(\Omega _1, K_1)\subseteq W^{s,p_1}(\Omega _1, {\mathbb {R}}^{M_1})$, then the functional :is coercive. This can be seen as follows:

The inequality between  and  resp.  and , see Assumption 2.1, carries over to  and , i.e.,for all $w \in W^{s,p_1}(\Omega _1, K_1)$.

Thus, it is sufficient to show that  is coercive: To prove this, we write shortly  instead of  and consider sequences $(w_n)_{n \in {\mathbb {N}}}$ in $W^{s,p_1}(\Omega _1, K_1)$ with  as . We show that , as . Since

$$\begin{aligned} \left\| w_n\right\| _{W^{s,p_1}(\Omega _1, {\mathbb {R}}^{M_1})}= & {} \left( \left\| w_n\right\| _{L^{p_1}(\Omega _1, {\mathbb {R}}^{M_1})}^{p_1} \right. \\&\left. \quad +\, \left| w_n\right| _{W^{s,p_1}(\Omega _1, {\mathbb {R}}^{M_1})}^{p_1} \right) ^{\frac{1}{p_1}} \end{aligned}$$

the two main cases to be considered are  and .

***Case 1***.

The inverse triangle inequality and the norm coercivity of , Eq. 3.4, give   . Therefore, also

**Fig. 1:**
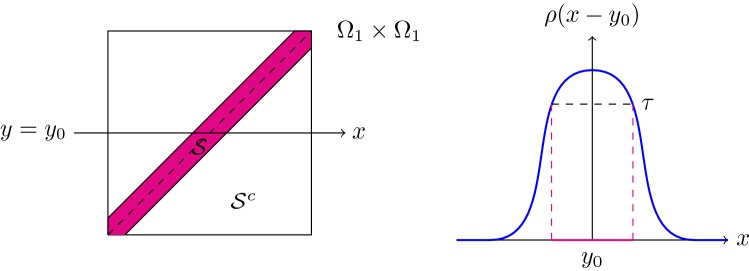
The stripe ${\mathcal {S}}= {\mathcal {S}}_{\tau }$ if $\Omega _1$ is an open interval and its connection to the radial mollifier $\rho$ for fixed $y \in \Omega _1$

***Case 2***.

If $l=0$, then  is exactly the $W^{s,p_1}(\Omega _1, {\mathbb {R}}^{M_1})$-semi-norm $|w|_{W^{s,p_1}(\Omega _1, {\mathbb {R}}^{M_1})}$ and we trivially get the desired result.

Hence, we assume from now on that $l = 1$. The assumptions on $\rho$ ensure that there exists a $\tau > 0$ and $\eta _{\tau }> 0$ such thatcf. Fig. 1.

Splitting $\Omega _1 \times \Omega _1$ into ${\mathcal {S}}_{\tau }=:{\mathcal {S}}$ and its complement $(\Omega _1 \times \Omega _1) \setminus {\mathcal {S}}_{\tau }=:{\mathcal {S}}^{c}$, we accordingly split the integrals  and consider again two cases  and , respectively.

***Case 2.1***.

By definition of ${\mathcal {S}}$ we have $\rho (x-y) \ge \tau > 0$ for all $(x,y) \in {\mathcal {S}}$. Therefore,Since $\alpha > 0$, it follows***Case 2.2***.

For $(x, y) \in {\mathcal {S}}^{c}$ it might happen that $\rho (x-y) = 0$, and thus instead of proving , as in Case 2.1, we rather show that . For this it is sufficient to show that for every $c > 0$ there is some $C \in {\mathbb {R}}$ such that the implicationholds true for all $w \in W^{s,p_1}(\Omega _1, K_1) \subseteq W^{s,p_1}(\Omega _1, {\mathbb {R}}^{M_1})$. To this end let $c > 0$ be given and consider an arbitrarily chosen $w \in W^{s,p_1}(\Omega _1, K_1)$ fulfilling .

Then . Using the triangle inequality and the monotonicity of the function $h: t \mapsto t^{p_2}$ on $[0, +\infty )$, we get further3.5Due to the norm coercivity, it thus follows that $\left\| w\right\| _{L^{p_1}(\Omega _1, {\mathbb {R}}^{M_1})} \le \bar{c}$, $\bar{c}$ some constant. Using [55, Lemma 3.20], it then follows that3.6for all $(x,y) \in \Omega _1 \times \Omega _1$. Using Eq. 3.6, Fubini’s Theorem and Eq. 3.5 we obtainCombining  for all $(x,y) \in {\mathcal {S}}^{c}$ with the previous inequality, we obtain the needed estimate

The second example concerns the coercivity of , defined in Eq. 2.9, when  denotes the *masking operator* occurring in image inpainting. To prove this result, we require the following auxiliary lemma:

#### Lemma 3.8

There exists a constant $C \in {\mathbb {R}}$ such that for all $w \in W^{s,p_1}(\Omega _1, {\mathbb {R}}^{M_1}), \ 0<s< 1, \ l \in \{0,1\}, \ 1< p_1 < \infty$ and $D \subsetneq \Omega _1$ nonempty such that3.7

#### Proof

The proof is inspired by the proof of Poincaré’s inequality in [29]. It is included here for the sake of completeness.

Assume first that $l=1$. Let ${\mathcal {S}}$ be as above,If the stated inequality Eq. 3.7 would be false, then for every $n \in {\mathbb {N}}$ there would exists a function $w_n \in W^{s,p_1}(\Omega _1, {\mathbb {R}}^{M_1})$ satisfying3.8By normalizing we can assume without loss of generality

(i) $$\left\| w_n\right\| _{L^{p_1}\left( D, {\mathbb {R}}^{M_1}\right) }^{p_1} = 1$$.

Moreover, by Eq. 3.8

(ii) $$\left\| w_n\right\| _{L^{p_1}(\Omega _1 \setminus D, {\mathbb {R}}^{M_1})}^{p_1} < \frac{1}{n}$$,
(iii).

By item *(i)* and item *(ii)*, we get that $\left\| w_n\right\| _{L^{p_1}(\Omega _1, {\mathbb {R}}^{M_1})}^{p_1} = \left\| w_n\right\| _{L^{p_1}\left( D, {\mathbb {R}}^{M_1}\right) }^{p_1} + \left\| w_n\right\| _{L^{p_1}(\Omega _1 \setminus D, {\mathbb {R}}^{M_1})}^{p_1}< 1 + \frac{1}{n} < 2$ is bounded. Moreoverwhere *c* is independent of *n*. This yields that the sequence $(w_n)_{n \in {\mathbb {N}}}$ is bounded in $W^{s,p_1}(\Omega _1, {\mathbb {R}}^{M_1})$ by $(2 + c)^{\frac{1}{p_1}}$. By the reflexivity of  for $p_1 \in (1, \infty )$ and Lemma 2.8, there exists a subsequence $(w_{n_k})_{k \in {\mathbb {N}}}$ of $(w_n)_{n \in {\mathbb {N}}}$ and $w_* \in W^{s,p_1}(\Omega _1, {\mathbb {R}}^{M_1})$ such that  strongly in $L^{p_1}(\Omega _1, {\mathbb {R}}^{M_1})$ and pointwise almost everywhere.

Using the continuity of the norm and dominated convergence, we obtain

(i) $\left\| w^*\right\| _{L^{p_1}\left( D, {\mathbb {R}}^{M_1}\right) }^{p_1} = 1$, in particular, $w^*$ is not the null function on D,
(ii) $\left\| w^*\right\| _{L^{p_1}(\Omega _1 \setminus D, {\mathbb {R}}^{M_1})}^{p_1} = 0$ since $n \in {\mathbb {N}}$ is arbitrary and hence $w^* \equiv 0$ on $\Omega _1 \setminus D$.
(iii) i.e., $$w^*(x) = w^*(y) $$ for $$(x,y) \in {\mathcal {S}}$$ yielding that $$w^*$$ locally constant and hence even constant since $$\Omega _1$$ is connected,

which gives the contradiction.

In the case $l=0$ we use similar arguments, where the distance  in the last inequality can be estimated by $\text {diam}|\Omega _1|$ (instead of $\eta$) since $\Omega _1$ is bounded. $\square$

#### Remark 3.9

In case $l=1$ it follows that the sharper inequality holds true: There exists a constant $C \in {\mathbb {R}}$ such that for all $w \in W^{s,p_1}(\Omega _1, {\mathbb {R}}^{M_1}), \ 0<s< 1, \ 1< p_1 < \infty$ and $D \subsetneq \Omega _1$ nonempty such that3.9

#### Example 3.10

As in Example 3.7, we consider that $W(\Omega _1,K_1) = W^{s, p_1}(\Omega _1, K_1)$ with $p_1>1, \ 0< s < 1$ and fix $k = N$.

Assume that  is the inpainting operator, i.e.,where $D \subseteq \Omega _1, \ w \in W^{s,p_1}(\Omega _1, K_1)$. Since the dimension of the data *w* and the image data  has the same dimension at every point $x \in \Omega _1$, we write $M :=M_1 = M_2$.

Then the functional :is coercive for $p_2 \ge p_1$:

The fact that $p_2 \ge p_1$ and that $\Omega _1$ is bounded ensures that

3.10 $$\begin{aligned} L^{p_2}(\Omega _1 \backslash D, {\mathbb {R}}^M) \subseteq L^{p_1}(\Omega _1 \backslash D, {\mathbb {R}}^M). \end{aligned}$$

The proof is done using the same arguments as in the proof of Example 3.7, where we additionally split ***Case 1*** into the two subcases

***Case 1.1***

***Case 1.2***

and using additionally Lemma 3.8, Eqs. 3.9 and 3.10.

## Stability and Convergence

In this section we will first show a stability and afterwards a convergence result. We use the notation introduced in Sect. 2. In particular, $W(\Omega _1, K_1)$ is as defined in Eq. 2.5. We also stress that we use notationally simplified versions  of  and ${\mathcal {R}}$ of  whenever possible. See Eqs. 2.6, 2.7 and 2.8.

### Theorem 4.1

Let Assumption 3.2 be satisfied. Let $v^\delta \in L^{p_2}(\Omega _2, K_2)$ and let $(v_n)_{n \in {\mathbb {N}}}$ be a sequence in $L^{p_2}(\Omega _2, K_2)$ such that . Then every sequence  withhas a converging subsequence w.r.t. the topology of $W(\Omega _1, K_1)$. The limit $\tilde{w}$ of any such converging subsequence $(w_{n_k})_{k \in {\mathbb {N}}}$ is a minimizer of . Moreover, $({\mathcal {R}}(w_{n_k}))_{k \in {\mathbb {N}}}$ converges to ${\mathcal {R}}(\tilde{w})$.

The subsequent proof of Theorem 4.1 is similar to the proof of [55, Theorem 3.23].

### Proof

For the ease of notation, we simply write  instead of  and .

By assumption the sequence  converges to 0 and thus is bounded, i.e., there exists $B \in (0, +\infty )$ such that4.1Because  it follows that4.2By Assumption 3.2 there is a $\overline{w} \in W(\Omega _1, K_1)$ such that . Set $c :=2^{p_2-1}$. Using Assumption 3.2 and applying Lemma 3.5, Eqs.  4.2 and 4.1 implies that for all $n \in {\mathbb {N}}$Applying again Lemma 3.5, we obtain . Hence, from item (3.1) it follows that the sequence  contains a converging subsequence.

Let now $(w_{n_k})_{k \in {\mathbb {N}}}$ be an arbitrary subsequence of  which converges in $W(\Omega _1, K_1)$ to some ${\tilde{w}} \in W(\Omega _1, {\mathbb {R}}^{M_1})$. Then, from Lemma 2.8 and the continuity properties of  it follows that ${\tilde{w}} \in W(\Omega _1, K_1)$ and  in $L^{p_2}(\Omega _2, K_2) \times L^{p_2}(\Omega _2, K_2)$. Moreover, using Lemma 3.4, Eq. 4.2 and the triangle inequality it follows that for every $w \in W(\Omega _1, K_1)$ the following estimate holds trueThis shows that ${\tilde{w}}$ is a minimizer of . Choosing $w = {\tilde{w}}$ in the previous estimate, we obtain the equalityDue to  this gives$\square$

Before proving the next theorem, we need the following definition, cf. [55].

### Definition 4.2

Let . Every element $w^* \in W(\Omega _1, K_1)$ fulfilling4.3is called an ${\mathcal {R}}$-*minimizing solution* of the equation  or shorter just ${\mathcal {R}}$-*minimizing solution*.

The following theorem and its proof are inspired by [55, Theorem 3.26].

### Theorem 4.3

Let Assumption 3.2 be satisfied. Let there exist an ${\mathcal {R}}$-minimizing solution $w^\dagger \in W(\Omega _1, K_1)$ and let  be a function satisfying4.4Let $(\delta _n)_{n \in {\mathbb {N}}}$ be a sequence of positive real numbers converging to 0. Moreover, let $(v_n)_{n \in {\mathbb {N}}}$ be a sequence in $L^{p_2}(\Omega _2, K_2)$ with  and set $\alpha _n :=\alpha (\delta _n)$.

Then every sequence  of minimizershas a converging subsequence  as $k \rightarrow \infty$, and the limit $\tilde{w}$ is always an ${\mathcal {R}}$-minimizing solution. In addition, .

Moreover, if $w^\dagger$ is unique, it follows that  and .

### Proof

We write shortly  for . Taking into account that  it follows thatyielding  as . The triangle inequality gives  as  and Remark 3.1 ensures  as , so that4.5Sincewe also get4.6Set $\alpha _{\text {max}} :=\max \{\alpha _n : n \in {\mathbb {N}}\}$. Sincethe sequence  is bounded. From Assumption 3.2, item (3.1) it follows that there exists a converging subsequence $(w_{n_k})_{k \in {\mathbb {N}}}$ of . The limit of $(w_{n_k})_{k \in {\mathbb {N}}}$ is denoted by $\tilde{w}$. Then, from Lemma 2.8 it follows that $\tilde{w} \in W(\Omega _1, K_1)$. Since the operator  is sequentially continuous, it follows that  in $L^{p_2}(\Omega _2, K_2)$. This shows that actually  since Eq. 4.5 is valid. Then, from Lemma 3.4 it follows that the functional  is sequentially lower semi-continuous, so that . Combining this with Eq. 4.6, we also obtainusing the definition of $w^\dagger$. This, together with the fact that  we see that $\tilde{w}$ is an ${\mathcal {R}}$-minimizing solution and that .

Now assume that the solution fulfilling Eq. 4.3 is unique; we call it $w^\dagger$. In order to prove that , it is sufficient to show that any subsequence has a further subsequence converging to $w^\dagger$, cf. [55, Lemma 8.2]. Hence, denote by $(w_{n_k})_{k \in {\mathbb {N}}}$ an arbitrary subsequence of $(w_n)$, the sequence of minimizers. Like before we can show that  is bounded and we can extract a converging subsequence $(w_{n_{k_l}})_{l \in {\mathbb {N}}}$. The limit of this subsequence is $w^\dagger$ since it is the unique solution fulfilling Eq. 4.3, showing that . Moreover, $w^\dagger \in W(\Omega _1, K_1)$. Following the arguments above, we obtain as well $\square$

### Remark 4.4

Theorem 4.1 guarantees that the minimizers of  depend continuously on $v^\delta$, while Theorem 4.3 ensures that they converge to a solution of , $v^0$ the exact data, while $\alpha$ tends to zero.

## Discussion of the Results and Conjectures

In this section we summarize some open problems related to double integral expressions of functions with values on manifolds.

### Relation to Single Integral Representations

In the following we show for one particular case of functions that have values in a manifold, that the double integral formulation , defined in Eq. 2.8, approximates a single energy integral. The basic ingredient for this derivation is the exponential map related to the metric $d_1$ on the manifold. In the following we investigate manifold-valued functions $w \in W^{1,2}(\Omega , \mathcal {M})$, where we consider $\mathcal {M} \subseteq {\mathbb {R}}^{M \times 1}$ to be a connected, complete Riemannian manifold. In this case some of the regularization functionals , defined in Eq. 2.8, can be considered as approximations of *single* integrals. In particular, we aim to generalize Eq. 1.3 in the case $p=2$.

We have that

$$\begin{aligned} \nabla w = \begin{bmatrix} \frac{\partial w_1}{\partial x_1}&\cdots&\frac{\partial w_1}{\partial x_N} \\ \vdots&\ddots&\vdots \\ \frac{\partial w_M}{\partial x_1}&\cdots&\frac{\partial w_M}{\partial x_N} \end{bmatrix} \in {\mathbb {R}}^{M \times N}. \end{aligned}$$

In the following we will write  instead of  to stress the dependence on $\varepsilon$ in contrast to above; the factor $\frac{1}{2}$ was added due to reasons of calculation. Moreover, let $\hat{\rho } : {\mathbb {R}}_+ \rightarrow {\mathbb {R}}_+$ be in $C_c^\infty ({\mathbb {R}}_+, {\mathbb {R}}_+)$ and satisfy

$$\begin{aligned} \left| \mathbb {S}^{N-1}\right| \int _0^\infty \hat{t}^{N-1} \hat{\rho }\left( \hat{t}\right) d \hat{t} = 1\;. \end{aligned}$$

Then for every $\varepsilon > 0$is a mollifier, cf. Example 2.2.

 (with $p_1=2$) then reads as follows:5.1Substitution with spherical coordinates $y = x - t \theta \in {\mathbb {R}}^{N \times 1}$ with $\theta \in \mathbb {S}^{N-1} \subseteq {\mathbb {R}}^{N \times 1}$, $t \ge 0$ gives5.2Now, using that for $m_1 \in \mathcal {M}$ fixed and $m_2 \in \mathcal {M}$ such that $m_1$ and $m_2$ are joined by a unique minimizing geodesic (see, for instance, [30] where the concept of exponential mappings is explained)

5.3 $$\begin{aligned} \frac{1}{2} \partial _2 d_1^2(m_1,m_2) = - (\exp _{m_2})^{-1}(m_1) \in {\mathbb {R}}^{M \times 1}, \end{aligned}$$

where $\partial _2$ denotes the derivative of $d_1^2$ with respect to the second component. By application of the chain rule we get

$$\begin{aligned} \begin{aligned}&- \frac{1}{2} \nabla _y d_1^2(w(x),w(y)) \\&\quad = \underbrace{(\nabla w(y))^\mathrm{T}}_{\in {\mathbb {R}}^{N \times M}} \underbrace{(\exp _{w(y)})^{-1}(w(x))}_{\in {\mathbb {R}}^{M \times 1}}\in {\mathbb {R}}^{N \times 1}\;, \end{aligned} \end{aligned}$$

where *w*(*x*) and *w*(*y*) are joined by a unique minimizing geodesic. This assumption seems reasonable due to the fact that we consider the case $\varepsilon \searrow 0$. Let $\cdot$ denote the scalar multiplication of two vectors in ${\mathbb {R}}^{N \times 1}$, then the last equality shows that

$$\begin{aligned} \begin{aligned}&\frac{1}{2} d_1^2(w(x),w(x-t \theta ))\\&\quad = - \frac{1}{2} \left[ d_1^2\big (w(x),w( (x-t\theta ) + t \theta )\big ) \right. \\&\qquad \left. - \, d_1^2\big (w(x),w(x-t \theta )\big ) \right] \\&\quad \approx \left( \left( \nabla w(x-t \theta )\right) ^\mathrm{T} (\exp _{w(x-t \theta )})^{-1}(w(x)) \right) \cdot t\theta \;. \end{aligned} \end{aligned}$$

Thus, from Eq. 5.2 it follows that5.4Now we will use a Taylor series of power 0 for $t\mapsto \nabla w(x-t \theta )$ and of power 1 for $t \mapsto (\exp _{w(x-t \theta )})^{-1}(w(x))$ to rewrite Eq. 5.4. We write

5.5 $$\begin{aligned} F(w;x,t,\theta ) :=(\exp _{w(x-t \theta )})^{-1}(w(x)) \in {\mathbb {R}}^{M \times 1} \end{aligned}$$

and define

5.6 $$\begin{aligned} \dot{F}(w;x,\theta ):= & {} \lim _{t \searrow 0} \frac{1}{t} \left( (\exp _{w(x-t \theta )})^{-1}(w(x)) \right. \nonumber \\&\quad \left. -\, \underbrace{(\exp _{w(x)})^{-1}(w(x))}_{=0} \right) \in {\mathbb {R}}^{M \times 1}. \end{aligned}$$

Note that because $(\exp _{w(x)})^{-1}(w(x))$ vanishes, $\dot{F}(w(x);\theta )$ is the leading order term of the expansion of $(\exp _{w(x-t \theta )})^{-1}(w(x))$ with respect to *t*. Moreover, in the case that $\nabla w(x) \ne 0$ this is the leading order approximation of $\nabla w(x-t \theta )$. In summary we are calculating the leading order term of the expansion with respect to *t*.

Then from Eq. 5.4 it follows that5.7The previous calculations show that the double integral simplifies to a double integral where the inner integration domain has one dimension less than the original integral. Under certain assumption the integration domain can be further simplified:

#### Example 5.1

If , $$p_1=2$$, then

$$\begin{aligned} \dot{F}(w;x,\theta )= & {} \lim _{t \searrow 0} \frac{1}{t} \left( w(x) - w(x-t\theta )\right) \\= & {} \nabla w(x)\theta \in {\mathbb {R}}^{M \times 1}. \end{aligned}$$

Thus, from (5.7) it follows that5.8This is exactly the identity derived in Bourgain et al. [14].

From these considerations we can view  as functionals, which generalize Sobolev and $\text {BV}$ semi-norms to functions with values on manifolds.

### A Conjecture on Sobolev Semi-norms

Starting point for this conjecture is Eq. 2.8. We will write $\Omega ,M$ and *p* instead of $\Omega _1, M_1$ and $p_1$.

- In the case $l=0$, $k=N$, $0<s<1$ and  the functional  from Eq. 2.8 simplifies to the *p*-th power of the Sobolev semi-norm and reads 5.9 For a recent survey on fractional Sobolev spaces, see [25].
- On the other hand, when we choose $k=0$, $l=1$ and , then  from Eq. 2.8 reads (note $\rho =\rho _\varepsilon$ by simplification of notation): 5.10
- Therefore, in analogy to what we know for $s=1$ from [14], we conjecture that 5.11 The form Eq. 5.11 is numerically preferable to the standard Sobolev semi-norm Eq. 5.9, because $\rho =\rho _\varepsilon$ and thus the integral kernel has compact support.

## Numerical Examples

In this section we present some numerical examples for denoising and inpainting of functions with values on the circle ${\mathbb {S}}^1$. Functions with values on a sphere have already been investigated very diligently (see, for instance, [13] out of series of publications of these authors). Therefore, we review some of their results first.

### ${\mathbb {S}}^1$-Valued Data

Let $\emptyset \ne \Omega \subset {\mathbb {R}}$ or ${\mathbb {R}}^2$ be a bounded and simply connected open set with Lipschitz boundary. In [13] the question was considered when  can be represented by some function  satisfying

6.1 $$\begin{aligned} \Phi (u) :={\mathrm {e}}^{i u} = w. \end{aligned}$$

That is, the function *u* is a *lifting* of *w*.

#### Lemma 6.1

([13])

- Let $\Omega \subset {\mathbb {R}}$, $0< s < \infty$, $1< p < \infty$. Then for all  there exists  satisfying Eq. 6.1.
- Let $\Omega \subset {\mathbb {R}}^N$, $N \ge 2$, $0< s < 1$, $1< p < \infty$. Moreover, let $sp < 1$ or $sp \ge N$, then for all  there exists  satisfying Eq. 6.1.
  If $sp \in [1,N)$, then there exist functions  such that Eq. 6.1 does not hold with any function .

For6.2we consider the functional (note that by simplification of notation below $\rho =\rho _\varepsilon$ denotes a mollifier)6.3on , in accordance to Eq. 2.8.

Writing $w = \Phi (u)$ as in Eq. 6.1, we get the lifted functional6.4over the space .

#### Remark 6.2

- We note that in the case $k=0$, $s=1$ and $l=1$ these integrals correspond with the ones considered in Bourgain et al. [14] for functions with values on ${\mathbb {S}}^1$.
- If we choose $k=N$, $s=1$ and $l=0$, then this corresponds with Sobolev semi-norms on manifolds.
- Let $\varepsilon > 0$ fixed (that is, we consider neither a standard Sobolev regularization nor the limiting case $\varepsilon \rightarrow 0$ as in [14]). In this case we have proven coercivity of the functional  only with the following regularization functional, cf. Example 3.7 and Example 3.10:

We summarize a few results: The first lemma follows from elementary calculations:

#### Lemma 6.3

 and $\,{\mathrm {d}}_{{\mathbb {R}}^2}\big |_{{\mathbb {S}}^1\times {\mathbb {S}}^1}$ are equivalent.

#### Lemma 6.4

Let . Then .

#### Proof

This follows directly from the inequality $\Vert {\mathrm {e}}^{ia}-{\mathrm {e}}^{ib}\Vert \le \Vert a-b\Vert$ for all $a,b \in {\mathbb {R}}$. $\square$

Below we show that  is finite on .

#### Lemma 6.5

 maps  into $[0,\infty )$ (i.e., does not attain the value $+\infty$).

#### Proof

Let . Then by Lemma 6.4 we have that . Therefore, from Lemma 6.3 and Proposition 2.13 item (ii) it follows that . Hence, by definition, . $\square$

### Setting of Numerical Examples

In all numerical examples presented, we use a simplified setting with

$$\begin{aligned}&M_1 = M_2 =:M,\;K_1 = K_2 =:{\mathbb {S}}^1,\\&p_1 = p_2 =:p,\;k = N,\;l = 1, \end{aligned}$$

$\Omega _1 = \Omega _2 =:\Omega$ when considering image denoising, $\Omega _1 = \Omega$, $\Omega _2 = \Omega \setminus D$ when considering image inpainting, andAs a particular mollifier, we use $\rho _\varepsilon$ (see Example 2.2), which is defined via the one-dimensional normal distribution $\hat{\rho }(x) = \frac{1}{\sqrt{\pi }} {\mathrm {e}}^{-x^2}.$

### Regularization Functionals

Let  and  be as defined in Eqs. 6.3 and 6.4, respectively. In what follows, we consider the following regularization functional6.5on  and the lifted variant6.6over the space  (as in Sect. 6.1), where $\Phi$ is defined as in (6.1). Note that .

#### Lemma 6.6

Let $\emptyset \ne \Omega \subset {\mathbb {R}}$ or ${\mathbb {R}}^2$ be a bounded and simply connected open set with Lipschitz boundary. Let $1< p < \infty$ and $s \in (0,1)$. If $N=2$ assume that $sp < 1$ or $sp \ge 2$. Moreover, let Assumption 3.2 and Assumption 2.10 be satisfied. Then the mapping  attains a minimizer.

#### Proof

Let . Then by Lemma 6.4 we have that . As arguing as in the proof of Lemma 6.5, we see that .

Since we assume that Assumption 3.2 is satisfied, we get that  attains a minimizer . It follows from Lemma 6.1 that there exists a function $u^* \in W^{s,p}(\Omega , {\mathbb {R}})$ that can be lifted to $w^*$, i.e., $w^* = \Phi (u^*)$. Then $u^*$ is a minimizer of (6.6) by definition of  and $\Phi$. $\square$

### Numerical Minimization

In our concrete examples, we will consider two different operators . For numerical minimization we consider the functional from Eq. 6.6 in a discretized setting. For this purpose, we approximate the functions $u \in W^{s, p}(\Omega ,{\mathbb {R}})$, $0<s<1,1<p<\infty$ by quadratic B-spline functions and optimize with respect to the coefficients. We remark that this approximation is continuous and thus that sharp edges correspond to very steep slopes.

The noisy data $u^\delta$ are obtained by adding Gaussian white noise with variance $\sigma ^2$ to the approximation or the discretized approximation of *u*.

We apply a simple Gradient Descent scheme with fixed step length implemented in $\text {MATLAB}$.

### Denoising of ${\mathbb {S}}^1$-Valued Functions: The InSAR Problem

In this case the operator  is the inclusion operator. It is norm-coercive in the sense of Eq. 3.4 and hence Assumption 3.2 is fulfilled. For $\emptyset \ne \Omega \subset {\mathbb {R}}$ or ${\mathbb {R}}^2$ a bounded and simply connected open set, $1< p < \infty$ and $s \in (0,1)$ such that additionally $sp < 1$ or $sp \ge 2$ if $N=2$ we can apply Lemma 6.6 which ensures that the lifted functional  attains a minimizer $u \in W^{s, p}(\Omega ,{\mathbb {R}})$.

**Fig. 2:**
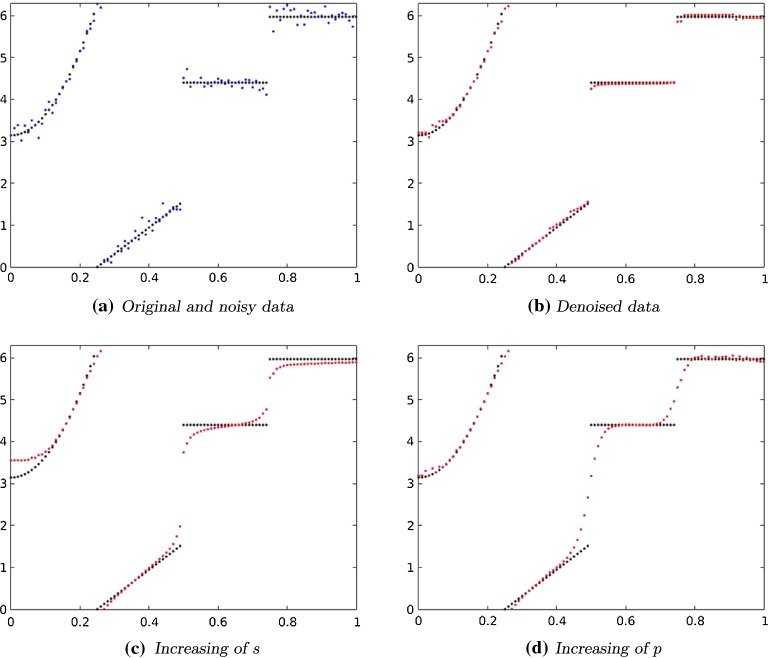
Function on ${\mathbb {S}}^1$ represented in $[0,2\pi )$: Left to right, top to bottom: original data (black) and noisy data (blue) with 100 data points. Denoised data (red) where we chose $s=0.1, p=1.1, \alpha = 0.19$. Denoised data with $s=0.6, p=1.1, \alpha = 0.19$ resp. $s=0.1, p=2, \alpha =0.19$ (Color figure online)

In the examples we will just consider the continuous approximation again denoted by *u*.

### One-Dimensional Test Case

Let $\Omega = (0,1)$ and consider the signal  representing the angle of a cyclic signal.

For the discrete approximation shown in Fig. 2a, the domain $\Omega$ is sampled equally at 100 points. *u* is affected by an additive white Gaussian noise with $\sigma = 0.1$ to obtain the noisy signal which is colored in blue in Fig. 2a.

**Fig. 3:**
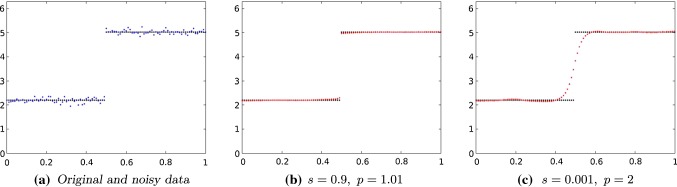
Left to right: original data (black) and noisy data (blue) sampled at 100 data points. Denoised data (red) where we chose $s=0.9, p=1.01, \alpha = 0.03$. Denoised data with $s=0.001, p=2, \alpha = 0.9$ (Color figure online)

In this experiment we show the influence of the parameters *s* and *p*. In all cases the choice of the regularization parameter $\alpha$ is 0.19 and $\varepsilon = 0.01$.

The red signal in Fig. 2b is obtained by choosing $s = 0.1$ and $p = 1.1$. We see that the periodicity of the signal is handled correctly and that there is nearly no staircasing. In Fig. 2c the parameter *s* is changed from 0.1 to 0.6. The value of the parameter *p* stays fixed. Increasing of *s* leads the signal to be more smooth. We can observe an even stronger similar effect when increasing *p* (here from 1.1 to 2) and letting *s* fixed, see Fig. 2d. This fits the expectation since *s* only appears once in the denominator of the regularizer. At a jump, increasing of *s* leads thus to an increasing of the regularization term. The parameter *p* appears twice in the regularizer. Huge jumps are hence weighted even more.

In Fig. 3a we considered a simple signal with a single huge jump. Again it is described by the angular value. We proceeded as above to obtain the approximated discrete original data (black) and noisy signal with $\sigma = 0.1$ (blue). We chose again $\varepsilon = 0.01$.

As we have seen above, increasing of *s* leads to a more smooth signal. This effect can be compensated by choosing a rather small value of *p*, i.e., $p \approx 1$. In Fig. 3b the value of *s* is 0.9. We see that it is still possible to reconstruct jumps by choosing, e.g., $p=1.01$.

**Fig. 4:**
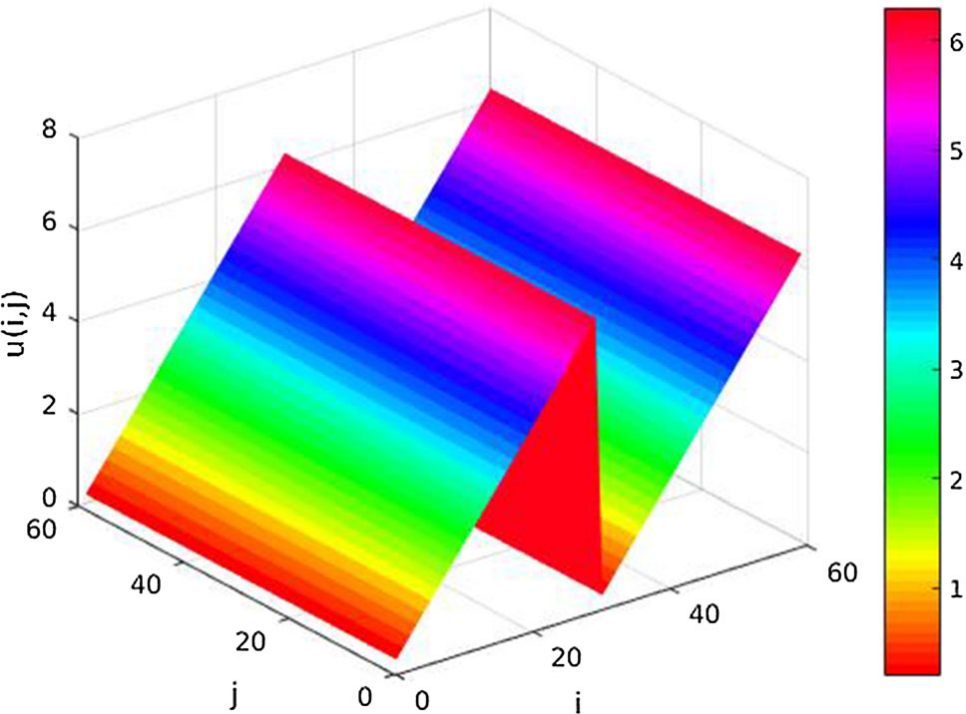
The function *u* evaluated on the discrete grid

**Fig. 5:**
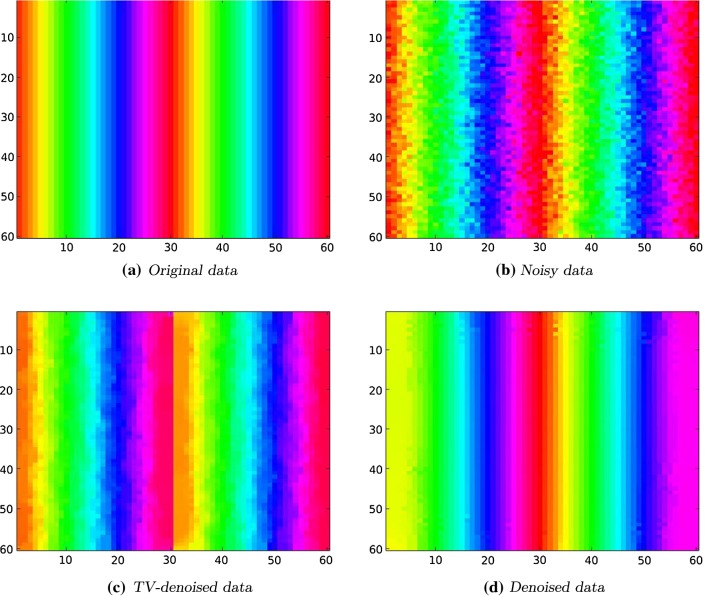
Left to right, top to bottom: original and noisy data of an $60 \times 60$ image. TV-denoised data using a fixed point iteration method. Denoised data where we chose $s=0.9, p=1.1, \alpha = 1$, 400 steps

Moreover, we have seen that increasing of *p* leads to an even more smooth signal. In Fig. 3c we choose a quite large value of *p*, $p=2$ and a rather small value of *s*, $s = 0.001$. Even for this very simple signal, it was not possible to get sharp edges. This is due to the fact that the parameter *p* (but not *s*) additionally weights the height of jumps in the regularizing term.

### Denoising of a ${\mathbb {S}}^1$-Valued Image

Our next example concerned a two-dimensional ${\mathbb {S}}^1$-valued image represented by the corresponding angular values. We remark that in this case where $N=2$ the existence of such a representation is always guaranteed in the cases where $sp < 1$ or $sp \ge 2$, see Lemma 6.1.

The domain $\Omega$ is sampled into $60 \times 60$ data points and can be considered as discrete grid, $\{1, \dots ,60\} \times \{1, \dots ,60\}$. The B-spline approximation evaluated at that grid is given by

$$\begin{aligned} u(i,j) = u(i,0) :=4\pi \frac{i}{60} \bmod 2\pi , \quad i,j \in \{1, \dots ,60\}. \end{aligned}$$

The function *u* is shown in Fig. 4. We used the $\text {hsv}$ colormap provided in $\text {MATLAB}$ transferred to the interval $[0, 2\pi ]$.

**Fig. 6:**
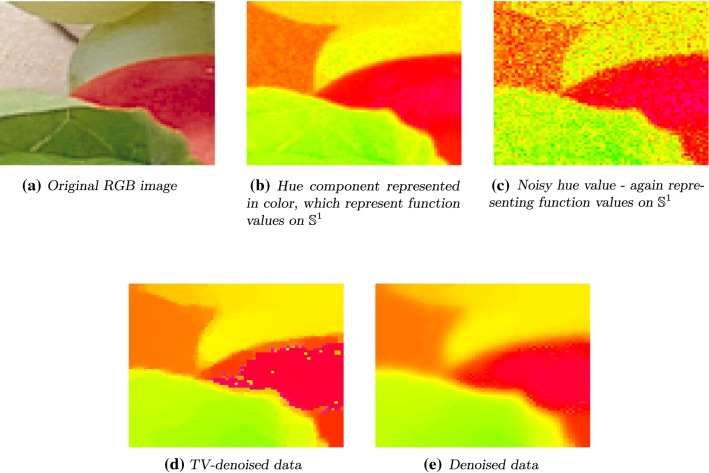
Left to right, top to bottom: original RGB image and its hue component. Noisy hue data with $\sigma ^2 = 0.001$. TV minimization is done using an iterative approach. It is serving as starting point for the GD minimization. Denoised data with $s=0.49, p=2, \alpha = 2$, 500 steps

**Fig. 7:**
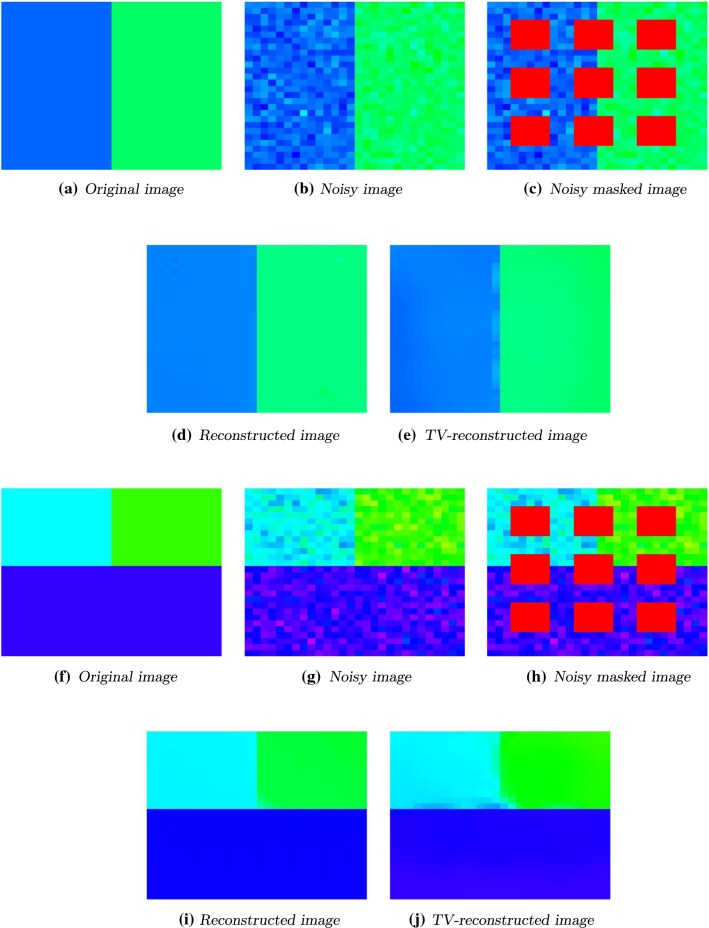
Left to right. Top to bottom: original image and the noisy data with $\sigma ^2 = 0.001$. Noisy image with masking filter and denoised data with $s=0.3, p=1.01, \alpha = 0.3$, 6000 steps. TV-denoised data. Original image and the noisy data with $\sigma ^2 = 0.001$. Noisy image with masking filter and denoised data with $s=0.4, p=1.01, \alpha = 0.4$, 10000 steps. TV-denoised image

**Fig. 8:**
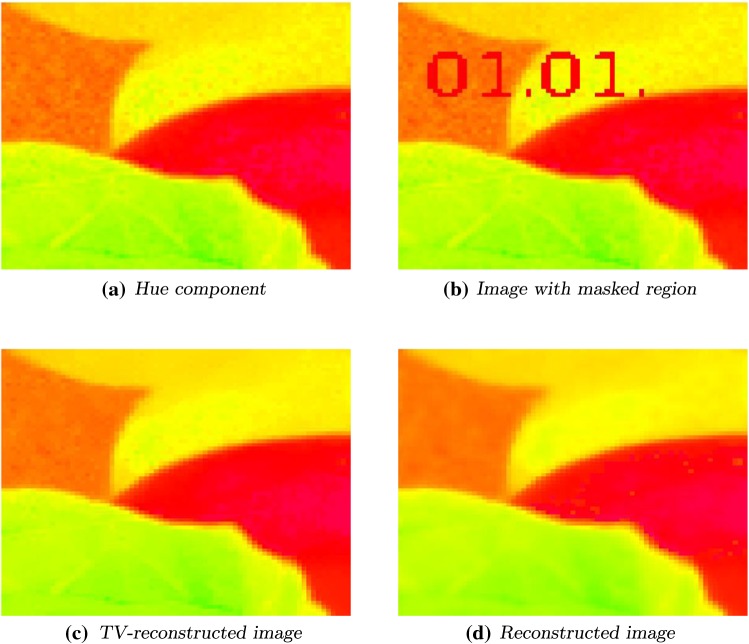
Left to right, top to bottom: original image and image with masked region. Reconstructed image with parameters $p=1.1, \ s=0.1, \ \alpha = 2$ and $\varepsilon = 0.006$, 2000 steps. TV-reconstructed image

This experiment shows the difference of our regularizer respecting the periodicity of the data in contrast to the classical total variation regularizer. The classical TV minimization is solved using a fixed point iteration ([45]); for the method see also [60].

In Fig. 5a the function *u* can be seen from the top, i.e., the axes correspond to the *i* resp. *j* axis in Fig. 4. The noisy data are obtained by adding white Gaussian noise with $\sigma = \sqrt{0.001}$ using the built-in function $\texttt {imnoise}$ in $\text {MATLAB}$. It is shown in Fig. 5b. We choose as parameters $s=0.9, \ p=1.1, \ \alpha = 1,$ and $\varepsilon = 0.01$. We observe significant noise reduction in both cases. However, only in Fig. 5d the color transitions are handled correctly. This is due to the fact that our regularizer respects the periodicity, i.e., for the functional there is no jump in Fig. 4 since 0 and $2\pi$ are identified. Using the classical TV regularizer, the values 0 and $2\pi$ are not identified and have a distance of $2\pi$. Hence, in the TV-denoised image there is a sharp edge in the middle of the image, see Fig. 5c.

### Hue Denoising

The $\text {HSV}$ color space is shorthand for Hue, Saturation, Value (of brightness). The hue value of a color image is ${\mathbb {S}}^1$-valued, while saturation and value of brightness are real-valued. Representing colors in this space better match the human perception than representing colors in the RGB space.

In Fig. 6a we see a part of size $70 \times 70$ of the RGB image “fruits” (https://homepages.cae.wisc.edu/~ece533/images/).

The corresponding hue data are shown in Fig. 6b, where we used again the colormap HSV, cf. Fig. 4. Each pixel value lies, after transformation, in the interval $[0, 2\pi )$ and represents the angular value. Gaussian white noise with $\sigma = \sqrt{0.001}$ is added to obtain a noisy image, see Fig. 6c.

To obtain the denoised image, in Fig. 6d we again used the same fixed point iteration, cf. [45], as before.

We see that the denoised image suffers from artifacts due to the non-consideration of periodicity. The pixel values in the middle of the apple (the red object in the original image) are close to $2\pi$ while those close to the border are nearly 0, meaning they have a distance of around $2\pi$.

We use this TV-denoised image as starting image to perform the minimization of our energy functional. As parameters we choose $s = 0.49, \ p = 2, \ \alpha = 2, \ \varepsilon = 0.006$.

Since the cyclic structure is respected, the disturbing artifacts in image in Fig. 6d are removed correctly. The edges are smoothed due to the high value of *p*, see Fig. 6e.

### ${\mathbb {S}}^1$-Valued Image Inpainting

In this case the operator  is the inpainting operator, i.e.,where $D \subseteq \Omega$ is the area to be inpainted.

We consider the functionalon .

According to Example 3.10, the functional  is coercive and Assumption 3.2 is satisfied. For $\emptyset \ne \Omega \subset {\mathbb {R}}$ or ${\mathbb {R}}^2$ a bounded and simply connected open set, $1< p < \infty$ and $s \in (0,1)$ such that additionally $sp < 1$ or $sp \ge 2$ if $N=2$ Lemma 6.6 applies which ensures that there exists a minimizer $u \in W^{s, p}(\Omega ,{\mathbb {R}})$ of the lifted functional $u \in W^{s, p}(\Omega ,{\mathbb {R}})$

### Inpainting of a ${\mathbb {S}}^1$-Valued Image

As a first inpainting test example, we consider two ${\mathbb {S}}^1$-valued images of size $28 \times 28$, see Fig. 7, represented by its angular values. In both cases the ground truth can be seen in Fig. 7a, f. We added Gaussian white noise with $\sigma = \sqrt{0.001}$ using the $\text {MATLAB}$ build-in function $\texttt {imnoise}$. The noisy images can be seen in Fig. 7b, g. The region *D* consists of the nine red squares in Fig. 7c, h.

The reconstructed data are shown in Fig. 7d, i.

For the two-colored image, we used as parameters $\alpha = s = 0.3$, $p = 1.01$ and $\varepsilon = 0.05$. We see that the reconstructed edge appears sharp. The unknown squares, which are completely surrounded by one color, are inpainted perfectly. The blue and green color changed slightly.

As parameters for the three-colored image, we used $\alpha = s = 0.4$, $p=1.01$ and $\varepsilon = 0.05$. Here again the unknown regions lying entirely in one color are inpainted perfectly. The edges are preserved. Just the corner in the middle of the image is slightly smoothed.

In Fig. 7e, j the TV-reconstructed data are shown. The underlying algorithm ([31]) uses the split Bregman method (see [36]).

In Fig. 7e the edge is not completely sharp. There are some lighter parts on the blue side. This can be caused by the fact that the unknown domain in this area is not exactly symmetric with respect to the edge. This is also the case in Fig. 7j where we observe the same effect. Unknown squares lying entirely in one color are perfectly inpainted.

### Hue Inpainting

As a last example, we consider again the hue component of the image “fruits”, see Fig. 8a. The unknown region *D* is the string $\textit{01.01}$ which is shown in Fig. 8b. As parameters we choose $p=1.1$, $s=0.1$, $\alpha = 2$ and $\varepsilon = 0.006$. We get the reconstructed image shown in Fig. 8c. The edges are preserved and the unknown area is restored quite well. This can be also observed in the TV-reconstructed image in Fig. 8d, using again the split Bregman method as before, cf. [31].

### Conclusion

In this paper we developed a functional for regularization of functions with values in a set of vectors. The regularization functional is a derivative-free, nonlocal term, which is based on a characterization of Sobolev spaces of *intensity data* derived by Bourgain, Brézis, Mironescu and Dávila. Our objective has been to extend their double integral functionals in a natural way to functions with values in a set of vectors, in particular functions with values on an embedded manifold. These new integral representations are used for regularization on a subset of the (fractional) Sobolev space $W^{s,p}(\Omega , {\mathbb {R}}^M)$ and the space $BV(\Omega , {\mathbb {R}}^M)$, respectively. We presented numerical results for denoising of artificial InSAR data as well as an example of inpainting. Moreover, several conjectures are at hand on relations between double metric integral regularization functionals and single integral representations.
